# Diversity within *Aspergillus niger* Clade and Description of a New Species: *Aspergillus vinaceus* sp. nov.

**DOI:** 10.3390/jof6040371

**Published:** 2020-12-17

**Authors:** Josué J. da Silva, Beatriz T. Iamanaka, Larissa S. Ferranti, Fernanda P. Massi, Marta H. Taniwaki, Olivier Puel, Sophie Lorber, Jens C. Frisvad, Maria Helena P. Fungaro

**Affiliations:** 1 Centro de Ciências Biológicas, Universidade Estadual de Londrina, Londrina, Paraná 86057-970, Brazil; josue.biomol@gmail.com (J.J.d.S.); issaferranti@uol.com.br (L.S.F.); fernanda_pm87@hotmail.com (F.P.M.); 2 Centro de Ciência e Qualidade de Alimentos, Instituto de Tecnologia de Alimentos, Campinas, São Paulo 13070-178, Brazil; beatriz@ital.sp.gov.br (B.T.I.); marta@ital.sp.gov.br (M.H.T.); 3 Toxalim (Research Centre in Food Toxicology), INRAE, ENVT, INP-Purpan, 31027 Toulouse, France; olivier.puel@inrae.fr (O.P.); sophie.lorber@inrae.fr (S.L.); 4 Department of Biotechnology and Biomedicine, Technical University of Denmark, 2800 Lyngby, Denmark; jcf@bio.dtu.dk

**Keywords:** *Aspergillus niger* clade, GCPSR, genealogical concordance, genetic diversity, secondary metabolites

## Abstract

Diversity of species within *Aspergillus niger* clade, currently represented by *A. niger sensu stricto* and *A. welwitshiae*, was investigated combining three-locus gene sequences, *Random Amplified Polymorphic DNA*, secondary metabolites profile and morphology. Firstly, approximately 700 accessions belonging to this clade were investigated using calmodulin gene sequences. Based on these sequences, eight haplotypes were clearly identified as *A. niger* (*n* = 247) and 17 as *A. welwitschiae* (*n* = 403). However, calmodulin sequences did not provide definitive species identities for six haplotypes. To elucidate the taxonomic position of these haplotypes, two other *loci*, part of the beta-tubulin gene and part of the RNA polymerase II gene, were sequenced and used to perform an analysis of Genealogical Concordance Phylogenetic Species Recognition. This analysis enabled the recognition of two new phylogenetic species. One of the new phylogenetic species showed morphological and chemical distinguishable features in comparison to the known species *A. welwitschiae* and *A. niger*. This species is illustrated and described as *Aspergillus vinaceus* sp. nov. In contrast to *A. niger* and *A. welwitschiae*, *A. vinaceus* strains produced asperazine, but none of them were found to produce ochratoxin A and/or fumonisins. Sclerotium production on laboratory media, which does not occur in strains of *A. niger* and *A. welwitschiae,* and strictly sclerotium-associated secondary metabolites (14-Epi-hydroxy-10,23-dihydro-24,25-dehydroaflavinine; 10,23-Dihydro-24,25-dehydroaflavinine; 10,23-Dihydro-24,25-dehydro-21-oxo-aflavinine) were found in *A. vinaceus.* The strain type of *A. vinaceus* sp. nov. is ITAL 47,456 (T) (=IBT 35556).

## 1. Introduction

The black aspergilli are among the most important fungal groups in the genus *Aspergillus*. These aspergilli include species causing food spoilage and mycotoxin production, often being reported as the third most common *Aspergillus* spp. associated with invasive human disease and aspergillomas [1,2]. Conversely, this fungal group includes several of the most industrially important species. For instance, *A. niger* is used in the production of various enzymes and organic acids, including 99% of the citric acid produced worldwide (1.4 million tons per year [3]).

Taxonomically, black aspergilli are classified as *Aspergillus* subgenus *Circumdati* section *Nigri*, but traditional morphological approaches do not perform well for the identification of all species. The main difficulties are related to the identification of the cryptic species (i.e., morphologically similar species). Currently, gene sequence analysis is necessary to establish the taxonomic status of this section. The rDNA-ITS region, which is the official DNA barcode for fungi, is too conserved for distinguishing the species of the *Aspergillus* section *Nigri*; therefore, the calmodulin (*CaM)* gene sequence has been recommended as a secondary barcoding marker [4,5].

*At present, in light of molecular phylogeny*, 27 species are included in section Nigri which [5,6], in turn, is split into seven clades: *A. tubingensis*, *A. niger*, *A. brasiliensis*, *A. carbonarius*, *A. heteromorphus*, *A. homomorphus* and *A. aculeatus*. The species *A. costaricaensis*, *A. luchuensis*, *A. neoniger*, *A. piperis*, *A. tubingensis*, *A. eucalypticola* and *A. vadensis* (clade *A. tubingensis*), *A. niger* and *A. welwitschiae* (*A. niger* clade) and *A. brasiliensis* (*A. brasiliensis* clade) are morphologically highly similar and have been generally called *Aspergillus niger* aggregate [5]. Consequently, it is imperative to provide additional data for their correct identification.

During the last 10 years, our research group has isolated a large number of *A. niger* aggregate strains from different Brazilian foodstuffs, in which species belonging to the *A. niger* clade are prevalent [7,8,9,10,11,12,13,14]. Since the *CaM* sequence database is complete for all accepted species of *A. niger* aggregate and, from a practical point of view, Samson and co-authors [5] suggested the use of this gene as a temporary secondary identification marker in *Aspergillus*, we used this approach to identify our Brazilian isolates [8,9,11,13]. Most of the isolates were identified as *A. niger* or *A*. *welwitschiae*, but the identity of certain isolates remained ambiguous. This has led us to apply, in this paper, the criteria used by Genealogical Concordance Phylogenetic Species Recognition (GCPSR) to resolve species boundaries based on individual and combined analyses of three genes (*CaM*, *BenA* and *RPB2*). Data were combined with secondary metabolite profiles, together with *Random Amplified Polymorphic DNA* and morphological traits.

## 2. Materials and Methods

### 2.1. Origin of CaM Sequences

A total of 403 strains belonging to the *A. niger* clade used in the present study were previously isolated from Brazilian foodstuffs (coffee beans, yerba mate, onion bulbs, grape berries, brazil nuts) and identified by means of Multiplex Polymerase Chain Reaction (mPCR) or partial calmodulin gene sequencing by our research group.

A portion of the *CaM* gene was amplified and sequenced for all strains in both directions, forward and reverse, using the primer-pair designed by Hong et al. [15], with amplification conditions shown in Table 1, and the sequencing protocol described in Taniwaki et al. [16]. With the *CaM* sequences obtained, we created the “Brazilian Dataset of *CaM* Sequences”.

Additionally, using the keywords calmodulin, *Aspergillus niger*, *A. welwitschiae*, *A. awamori*, *A. foetidus* and *A. lacticoffeatus,* 424 *CaM* sequences belonging to the *A. niger* clade were retrieved from GenBank (12 July 2018). From this total, 132 *CaM* sequences were removed because they were very short or of low-quality, or because they represent deposits with obsolete definitions. With the remaining sequences (*n* = 292), we created the “GenBank Dataset of *CaM* Sequences.”

The Brazilian Dataset (*n* = 403) plus the GenBank Dataset (*n* = 292) consisted of 695 *CaM* gene sequences (Supplementary Table S1).

### 2.2. Diversity and Phylogenetic Relationships of CaM Haplotypes

All sequences (the Brazilian Dataset of *CaM* Sequences and the GenBank Dataset of *CaM* Sequences) were aligned using Multiple Sequence Comparison by Log- Expectation (MUSCLE) [18] in the MEGA 7 computational package [19].

The haplotype identification, haplotype diversity (Hd) and nucleotide diversity (π) were performed using the DNA sp5.10 computational package [20]. The phylogenetic relationships among the haplotypes were first examined using *CaM locus* sequences. The *CaM* sequences of up to 3 representatives (when available) from each of the haplotypes were aligned with homologous sequences from holotype/neotype strains of *A.* section *Nigri* species. The phylogenetic tree was constructed using a maximum likelihood method with the Kimura-2-parameter model [21] (previously calculated in MEGA7 and jModelTest2) with discrete Gamma distribution and invariant sites (G + I) with Subtree Pruning Regrafting (SPR level 5) and a very weak branch swap filter. Sequence identity was also confirmed using a GenBank Nucleotide Basic Local Alignment Search Tool (BLASTn) [22].

### 2.3. Sequencing Beta-Tubulin (BenA) and RNA Polymerase II (RPB2) Genes

To elucidate the taxonomic position of the haplotypes identified in the present study, two other *loci*, part of the beta-tubulin (*BenA*) and part of the RNA polymerase II (*RPB2*) genes, both considered alternative markers for *A.* section *Nigri* [5], were sequenced and used to compose the Genealogical Concordance Phylogenetic Species Recognition (GCPSR) analysis. Up to 10 strains (when available) were selected for each haplotype present in the Brazilian dataset. Unfortunately, the GCPSR step was not possible for haplotypes found exclusively in the GenBank dataset, as we did not have physical access to these strains, and there are no deposits on GenBank for all *loci* (*CaM, BenA* and *RPB2*) of representatives of these haplotypes (H19, H20, H22 and H23).

The primers used for the amplification of the *BenA* gene were those described by Glass and Donaldson [17], with amplification conditions shown in Table 1. The sequencing protocol was the same as that used by Taniwaki et al. [16].

The primer-pair used for the amplification of the *RPB2* gene was designed in the present study. Primer design involved the recovery of *RPB2* gene sequences from all species of the *A. niger* aggregate available in GenBank, the alignment of the sequences using MUSCLE in MEGA 7 and a visual search for regions conserved within and between species. The melting temperature, the formation of secondary structures and other parameters were analysed in silico using the Oligoanalyzer 3.1 (https://www.idtdna.com/calc/Analyzer/Home/Instructions). The primer sequences and the segment of the *RPB2* gene (≅720 bp) are shown in Supplementary Figure S1. PCR amplification was performed according to the conditions shown in Table 1. All PCR products were purified and sequenced, in forward and reverse directions, on an ABI 3500 XL genetic analyser.

### 2.4. Genealogical Concordance Phylogenetic Species Recognition—GCPSR

Genealogical Concordance Phylogenetic Species Recognition (GCPSR) is a practical approach to Phylogenetic Species Recognition (PSR). The GCPSR protocol adopted in this study was that found in Dettman et al. [23]. Sequence data of three *loci* (*CaM*, *BenA* and *RPB2*) were analysed individually and in combination.

Initially, we recognised the independent evolutionary lineages (IELs) based on the criteria of genealogical concordance and genealogical non-discordance [23]. According to the criterion of genealogical concordance, a clade must be present in most of the phylogenies analysed, that is, when analysing the phylogeny of each gene tree separately, the evolutionary lineage must be consistent in most of them, regardless of support levels (Bootstrap Percentage, BP, or Posterior Probabilities, PP).

For the application of the genealogical concordance criterion, maximum parsimony (MP) and Bayesian Inference (BI) trees were constructed for each *locus*, individually. The alignments were performed using MUSCLE in MEGA 7. The *maximum* parsimony trees based on these sequence alignments were created using MEGA 7. The heuristic search option with 1000 bootstraps and Tree Bisection Reconnection (TBR) branch swapping were used to infer the most parsimonious trees, and a Majority-Rule Consensus (MRC 50%) was generated [24].

A Bayesian Inference (BI) analysis was conducted in MrBayes 3.2.3 [25]. The most suitable model of sequence evolution for each dataset was selected based on the lowest Akaike Information Criterion (AIC) value in jModeltest2 [26]. For BI, the Markov Chain Monte Carlo (MCMC) algorithm was run for 5 × 10^6^ generations with a sample frequency of 100 and with 25% of trees removed for burn-in. Convergence diagnostics were monitored based on standard deviations of frequencies below 0.01. The consensus tree topology (MRC 50%) and the PP for the nodes were visualised using FigTree version 1.4.3 (http://tree.bio.ed.ac.uk/software/figtree/).

According to the criterion of genealogical non-discordance, a clade can be considered to be an IEL if it is statistically supported (BP and PP) in at least one phylogeny and is not contradicted at the same level of support by another *locus*. The identification of these clades is done through analysis of a semi-strict consensus tree combining the phylogenies of all analysed *loci*. Dettman et al. [23] stipulated an arbitrary value of statistical support as 70% for BP values and 0.95 for PP.

For the application of the criterion of genealogical non-discordance, semi-strict consensus trees (MP and BI) were constructed. The semi-strict consensus trees were constructed in the PAUP 4.0 [27] based on the phylogenies of the three *loci* analysed.

The framing on one of the two criteria (genealogical concordance/non-discordance) satisfies the condition for the recognition of an IEL. To determine an IEL as a phylogenetic species, we used the criteria of genetic differentiation and exhaustive subdivision [23].

For analysis of genetic differentiation and exhaustive subdivision, maximum parsimony and Bayesian trees were constructed with the combined data, as established in the Dettman protocol. Additionally, we also built a tree of maximum likelihood with the same combined dataset. The combined dataset was partitioned into three blocks (*CaM, BenA* and *RPB2*) in the Partitionfinder2 on XSEDE platform (v2.1.1) [28]. The best model for the *CaM* block (430 bp) was K80 + G (BI) and GTR + G + I (ML), that for the *BenA* block (453 bp) was K80 + G (BI) and K80 (ML) and that for the *RPB2* block (625 bp) was GTR (BI) and K80 (ML). The programs used to construct the trees were MrBayes 3.2.3, RaxML v.8 on XSEDE [29] and PAUP 4.0.

In genetic differentiation, minor tip clades recognised as IELs must be relatively distinct and well-differentiated to avoid minor tip clades being recognised as phylogenetic species. In the exhaustive subdivision, all individuals must be assigned to a phylogenetic species. Therefore, if an individual was not included within an IEL, we collapsed this clade to the adjacent node, and this was done hierarchically until all the individuals were allocated within an IEL and the rankings possibilities are exhausted. The final groups were recognised as phylogenetic species.

### 2.5. Random Amplified Length Polymorphism (RAPD)

Random Amplified Polymorphic DNA (RAPD) analysis was applied to four phylogenetic species (7 strains representing each phylogenetic species). DNA amplifications were carried out using six arbitrary primers (OPAM02, OPAM07, OPAM10, OPAM11, OPAM13 and OPX11) in a Veriti^®^ Thermal Cycler (Applied Biosystems, Waltham, MA-USA). The conditions of the reactions were the same as those described in Fungaro et al. [30]. Amplified fragments were scored 1 for the presence and 0 for the absence in each strain. The program Ntsys-PC was used to obtain a similarity matrix using a Dice coefficient and a dendrogram with the Unweighted Pair Group Method with Arithmetic Mean (UPGMA). The allele frequencies of the sets of strains were estimated and submitted to pairwise *φst* analysis using Arlequin 3.5.2.2 [31].

Bayesian clustering was also used to assess genetic structuring of fungal groups using the program Structure 2.3.4 [32]. The number of clusters (*K*) was estimated using the admixture ancestral model, with *K* ranging from 1 to 8. Twenty independent runs of 10^5^ Markov Chain Monte Carlo (MCMC) generations with 2 × 10^4^ generations of “burn-in” were used for each value of *K.* To interpret the number of population clusters, *∆K* [33], statistics built into the Structure harvester [34] virtual platform were used.

### 2.6. Morphological Analysis

*The morphological analyses* were conducted in accordance with the guidelines of Samson et al. [5]. For macromorphological observations, representatives from each phylogenetic species identified by GCPSR (PS1, PS2, PS3 and PS4) were inoculated in three points onto Czapek Yeast Autolysate agar (CYA), Malt Extract Agar (MEA), Creatine Sucrose agar (CREA) and Yeast Extract Sucrose Agar (YESA) and incubated for 7 days at 25 °C in the dark. In addition, for growth pattern examination, CYA plates were incubated at 37 and 42 °C. The *experiments* were *performed* in triplicate.

For micromorphological observations (optical microscopy), microscopic mounts were made in lactic acid from MEA colonies (7 days at 25 °C). The microstructures: conidiophores, stipes, vesicles, conidia, metulae and phialides, were measured using the software AxioVision Release 4.8.2.

For scanning electron microscopy (SEM), 0.5 × 0.5 cm plugs (MEA, 7 days at 25 °C) were fixed in 2% glutaraldehyde solution at 4 °C, for 24 h, and later washed in 0.1 M sodium phosphate buffer, (three times, for 15 min). Subsequently, the plugs were post-fixed with 1% osmium tetroxide (aqueous solution) at room temperature for 2 h at 25 °C in the dark. After washing in 0.1 M sodium phosphate buffer, the fragments were dehydrated with ethanol at 70%, 80%, 90% and 100% for 10 min at each step. The samples were transferred to a critical-point dryer (Bal-Tec, CSDC 030) and gold-coated on a vaporiser (Bal-Tec, SDC050). The images were obtained with a Quanta 200^®^ (Field Electron and Ion Company-FEI, Hillsboro, OR, USA) Scanning Microscope.

### 2.7. Secondary Metabolites Characterisation of PS3 Strains

Ten PS3 strains were analysed for secondary metabolites by extracting 3 agar plugs from 7 days incubated cultures on CYA and YESA at an incubation temperature of 25 °C in darkness. The extracts were analysed by ultra-high performance liquid chromatography with diode array detection (UHPLC-DAD) and an acetonitrile/water gradient (both eluents containing 0.05% trifluoroacetic acid) solution as mobile phase and compared to authentic small-molecule extrolite standards as previously described by Frisvad and Thrane [35] and Nielsen et al. [36].

### 2.8. Secondary Metabolites Characterisation of Sclerotia in PS3 Strains

For secondary metabolites characterisation of sclerotia, the strain ITAL 47.456 was grown on MEA for 10 days at an incubation temperature of 25 °C in darkness. Sclerotia were recovered using the methodology described in Carvajal-Campos et al. [37]. Secondary metabolites were screened by liquid chromatography coupled to high-resolution mass spectrometry (HPLC-HRMS). The chromatographic system consisted of a RSLC3000 UHPLC (Thermo Scientific, Les Ulis, France) operating with a 5 µm Luna^®^ C18 column (125 × 2.0 mm Luna C18 5 µm column) (Thermo Scientific, Les Ulis, France). A gradient program of (A) water acidified with 0.05% formic acid and (B) acetonitrile was used at 30 °C and a flow rate of 0.2 mL min^−1^ as follows: 0 min 20% B, 30 min 50% B, 35 min 90% B, from 35 to 45 min 90% B, 50 min 20% B, from 50 to 60 min 20% B. A volume of 10 µL of each sample diluted twice with mobile phase A was injected. The mass spectrometer corresponded to an LTQ Orbitrap XL (Thermo Scientific, Les Ulis, France) fitted with an Electrospray Ionisation Source (ESI). ESI parameters for the negative mode were set as follows: spray voltage: 3.7 kV, sheath gas flow rate (N_2_): 30 arbitrary units (a.u.), auxiliary gas flow rate (N_2_): 10 a.u., capillary temperature: 350 °C, capillary voltage: −34 V and tube lens offset: −180 V. ESI parameters for the positive mode were set as follows: spray voltage: 5.5 kV, sheath gas flow rate (N_2_): 30 arbitrary units (a.u.), auxiliary gas flow rate (N_2_): 10 a.u., capillary temperature: 350 °C, capillary voltage: 1 V and tube lens offset: 45 V. High-resolution mass spectra were acquired between *m/z* 80 and 800 at a resolution of 7500. The calibration of the mass spectrometer was achieved using the calibration solution of Thermo Scientific in agreement with their protocol. MS/MS spectra were obtained with the collision-induced dissociation (CID) mode of the ion trap analyser at low resolution and normalised collision energy of 35%.

## 3. Results and Discussion

### 3.1. Diversity and Phylogenetic Relationships of CaM Haplotypes

The 418-character matrix of the aligned *CaM* sequences generated from the Brazilian Dataset of *CaM* Sequences (*n* = 403) plus the GenBank Dataset of *CaM* Sequences (*n* = 292) enabled the detection of 47 variable sites with 18 parsimony informative sites, yielding 31 *CaM* haplotypes (Supplementary Table S1). Of the total number of haplotypes detected, 93.5% (29/31) were generated from Single Nucleotide Polymorphisms (SNP’s) and only 6.5% (2/31) were generated from Insertion or Deletion variants (INDEL’s).

Among all haplotypes, 19 were singletons, and 12 occurred from 2 to 336 times. The haplotype H8 (*n* = 336), which has an identical *CaM* sequence to that from the *A. welwitschiae* neotype strain (NRRL 4948), exhibited the highest frequency, and the haplotype H14 (*n* = 222), with a sequence identical to *A. niger* type strain (NRRL 326), was the second most frequent. This finding means that the haplotypes identical to *A. niger* and *A. welwitschiae* type strains accounted for 80.3% (558/695) of the total *CaM* sequences identified in the present study. Nine haplotypes (H2, H3, H4, H6, H7, H15, H25, H28, H30) were exclusively found in the Brazilian dataset and accounted for only 3.4% (24/695) of the total sequences. Conversely, seventeen haplotypes (H1, H5, H9, H10, H11, H12, H13, H16, H17, H18, H19, H20, H22, H23, H24, H26, H31) were exclusive to the GenBank dataset, accounting for 8.9% (62/695) of the total sequences (Supplementary Table S1).

**Figure 1:**
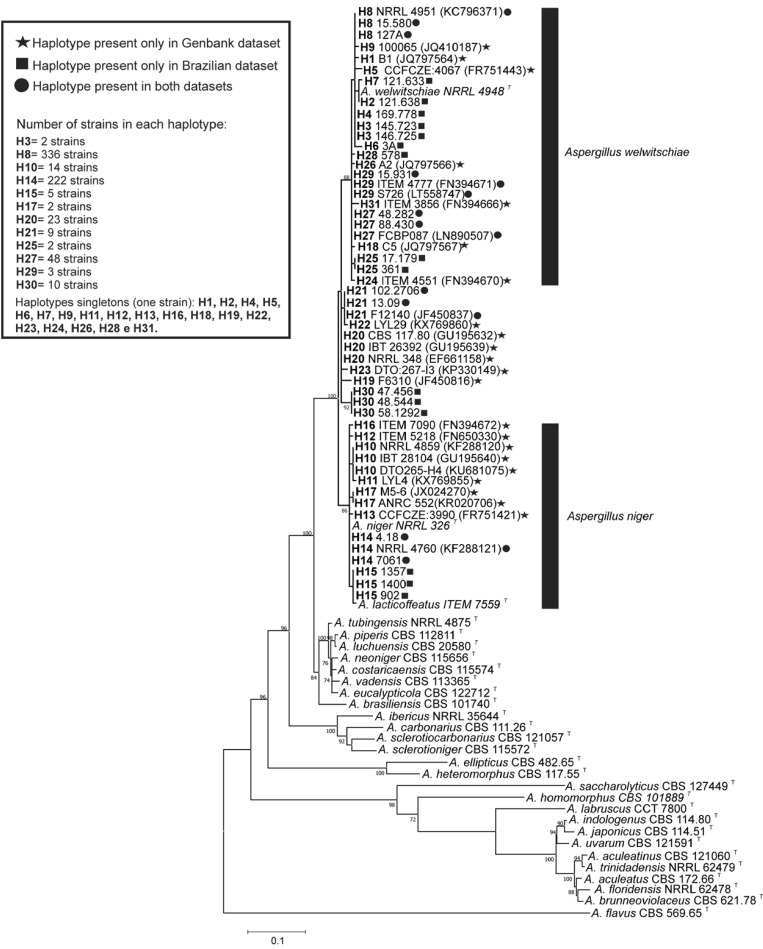
Maximum Likelihood tree of *Aspergillus* section *Nigri* species and *CaM* haplotypes of *A. niger* clade species. Only bootstrap ≥ 70% are shown. *Aspergillus flavus* is the outgroup. T superscript = Type strain.

By taking the 695 sequences as a single dataset, the overall haplotype diversity (Hd) and nucleotide diversity (π) were 0.654 ± 0.013 and 0.00990 ± 0.00100, respectively. When the Brazilian dataset (*n* = 403) and GenBank dataset (*n* = 292) sequences were analysed separately, higher diversity was noted in the GenBank dataset sequences (Hd = 0.686 ± 0.022; π = 0.00974 ± 0.00026) compared to the Brazilian dataset (Hd = 0.524 ± 0.025; π = 0.00762 ± 0.00044). The greater genetic diversity within GenBank dataset sequences is not surprising because GenBank receives sequences from strains isolated from different countries throughout the world and from a higher range of substrates than analysed here. The genetic relationships among haplotypes identified in the present study were inferred by phylogenetic analysis of the *CaM* nucleotide sequences. Most of the haplotypes are on well-supported branches with the neotype strain of *A. welwitschiae* (NRRL 4948) (98% bootstrap value) or with the type strain of *A. niger* (NRRL 326) (90% bootstrap value) and were confidently identified as *A. welwitschiae* (*n* = 403) or *A. niger* (*n* = 248), respectively (Figure 1). The exceptions to this identification were six haplotypes (H19, H20, H21, H22, H23, H30), representing 45 strains, which were not resolved on well-supported branches in the *A. niger* or *A welwitschiae* clades (Figure 1). Thus, the phylogenetic analyses using only *CaM* sequences did not provide definitive species identities for these haplotypes. Some authors have also found strains belonging to the *A. niger* clade with ambiguous identities [1,38].

Among the six haplotypes unresolved, only two of them (H30 and H21) occurred in the Brazilian dataset, and it was possible to investigate them more deeply to determine whether they might represent new phylogenetic species.

### 3.2. Genealogical Concordance Phylogenetic Species Recognition (GCPSR)

Genealogical Concordance Phylogenetic Species Recognition (GCPSR) is a term originally suggested by Taylor et al. [39] which reflects the concept of genealogical concordance established in Avise and Ball [40], later operationalised by Dettman et al. [23]. Briefly, it is an analysis of multiple *loci* realised by the observation of the phylogenies and the statistical support of the clades in two scenarios: individual and consensus genealogies.

For the GCPSR implementation, nucleotide sequences of 3 *loci* (*CaM, BenA* and *RPB2*) were used in our study. A total of 56 strains isolated from Brazilian foodstuffs were selected according to the analysis of haplotypes based on the *CaM locus* (secondary barcode for phylogeny of *A*. section *Nigri*) and additionally sequenced for parts of the *RPB2* and *BenA* genes. For GCPSR analysis, the alignment of the *CaM locus* resulted in a fragment of 430 bp (Hd = 0.863 ± 0.015; π = 0.01076 ± 0.00043), for the *BenA locus*, the final alignment resulted in 453 bp (Hd = 0.807 ± 0.024; π = 0.00395 ± 0.00024), and for the *RPB2 locus*, the final alignment resulted in 625 bp (Hd = 0.303 ± 0.067; π = 0.00340 ± 0.00075). As can be observed (Supplementary Figure S2), the *CaM locus* has more resolution power for phylogeny of the *A. niger* clade in relation to the other *loci* analysed. The combined data (*CaM + BenA + RPB2*) totalled 1508 bp (Hd = 0.880 ± 0.017; π = 0.00563 ± 0.00034).

Based on the criteria of genealogical concordance (Supplementary Figure S2) and genealogical non-discordance (Supplementary Figure S3), 8 independent evolutionary lineages (IELs) were recognised. IEL 1 and 2 were identified by the genealogical concordance criterion, and the remaining IELs were identified by the genealogical non-discordance criterion. As mentioned in the “Material and Methods Section” to recognise an IEL as a phylogenetic species (PS), the criteria of genetic differentiation and exhaustive subdivision [23] were used. The minor tip clades IEL 6 and 7 were excluded from the ranking process according to the genetic differentiation criterion.

**Figure 2:**
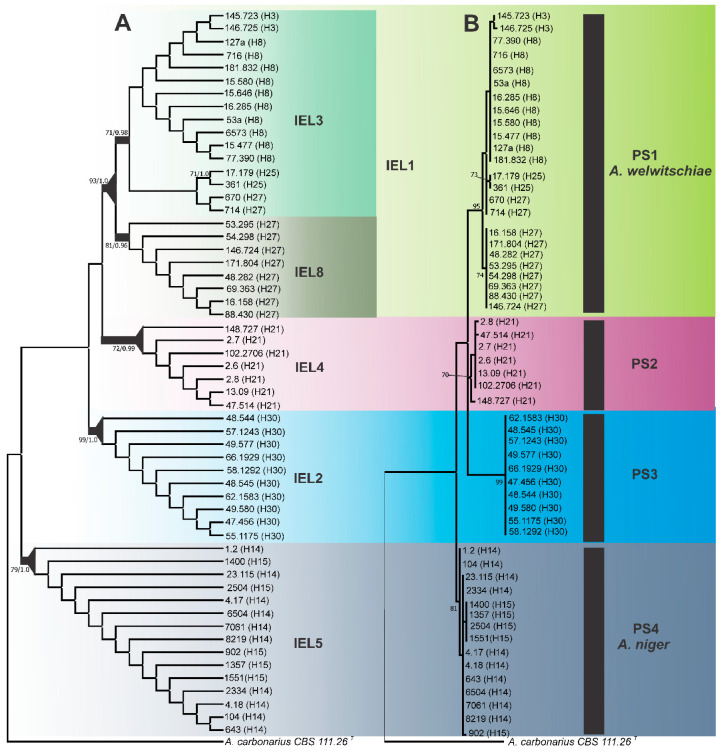
(**A**) Maximum Parsimony cladogram of *A. niger* clade species using three-*locus* (*CaM, BenA,* and *RPB2*) phylogenies. The branches in bold indicate that these clades were considered independent evolutionary lineages. The triangles at the nodes indicate phylogenetic species based on the ranking of groups (Dettman et al. [23]). Bootstrap values (BP) and/or posterior probabilities values (PP) higher than 70% and 0.70 respectively, are shown. *A. carbonarius* is the outgroup. (**B**) Maximum Likelihood tree based on concatenated *loci* (*CaM + BenA + RPB2*) of *Aspergillus niger* clade species. PS 2 = Phylogenetic species 2, PS3 = *Aspergillus vinaceus* sp. nov. Only bootstrap ≥ 70% are shown. *Aspergillus carbonarius* is the outgroup. Superscript T = Type strain.

The limits of the phylogenetic species were then established by means of the exhaustive subdivision criterion. Based on this criterion, IELs 3 and 8 were collapsed, and all individuals were harboured under IEL 1; therefore, this node was recognised as the limit of phylogenetic species 1 (PS1). A group of seven strains representing the IEL 4 formed the phylogenetic species 2 (PS2). This phylogenetic species was well-supported statistically in the combined three-locus datasets (Figure 2) and *CaM locus* analyses (Supplementary Figure S2).

The IEL 2 harboured 10 strains, and following the ranking process, it was identified as phylogenetic species 3 (PS3). This phylogenetic species was well-supported statistically in all analyses in the individual and/or combined genealogies (Figure 2 and Supplementary Figures S2 and S3).

Under the exhaustive subdivision criterion, the node of IEL 5, which was well-supported in the combined analysis (three-*locus* dataset), was recognised as a phylogenetic species boundary and designated as phylogenetic species 4 (PS4). Fifteen strains examined in this study were allocated to this phylogenetic species (Figure 2).

The strains belonging to the PS1 and PS4 were those identified as *A. welwitschiae* (H3, H8, H25, H27) and *A. niger* (H14 and H15), respectively. On the basis of our data, *A. welwitschiae* neotypes strains (NRRL 4948 and ITEM 4509) (*A. awamori sensu* Perrone) [41] are assigned to H8, whereas the exotype strain of *A. niger* (NRRL 326) is assigned to H14. It is worth mentioning that the type strains of *A. lacticoffeatus* (CBS 101883) and *A. foetidus* (NRRL 341) are also contained in Haplotype H14 (Supplementary Table S1), these species were previously synonymised to *A. niger stricto sensu.*

The PS2 (H21) and PS3 (H30) were not collapsed to *A. niger* and *A. welwitschiae*, thereby becoming candidates for new species.

Phylogenetic species 2 (PS2) was recognised by the sequence data collected from 7 Brazilian strains isolated from coffee beans, grape berries and onions bulbs. Based on *CaM* sequence, these seven strains (2.7, 2.6, 2.8, 47.514, 148.727, 102.2706, 13.09), as well as two clinical isolates (F8478 and F12140) characterised by Howard et al. [2] in their study of cryptic species in the *Aspergillus niger* complex, represent the haplotype H21 (Supplementary Table S1). There are five SNPs fixed for PS2 in relation to *A. niger* and four in relation to *A. welwitschiae.* Interestingly, among several *A. niger* and *A. welwitschiae* strains tested by Howard et al. [2], only the strain F12140 was highly cross-resistant to all azole drugs. Combining calmodulin and beta-tubulin sequencing, these authors observed that the F12140 isolate is most closely related to *A. awamori (currently A. welwitschiae)*; however, by the *cyp51A* sequence, the isolate was most similar to *A. niger*, although bootstrap values were low (<70) for all analyses. Using *cyp51A* sequences, the strain F12140 appears to be even more divergent from *A. awamori* and *A. niger* [2]. It is also important to note that the *CaM* sequence of the strain denoted as ITEM 1148 (see Susca et al. [38]) (sequence not available in GenBank), recovered from Brazilian cashew nuts, is identical to those from our seven Brazilian strains recognised as PS2 and that the identity of this strain was considered ambiguous by Susca et al. [38].

PS3 (=*Aspergillus vinaceus* sp. nov.), represented by haplotype H30 (Supplementary Table S1), was exclusive to the Brazilian dataset on 12 July 2018. All strains were isolated from grape samples grown in the São Francisco Valley, Northeastern Brazil (Supplementary Table S1). However, on 22 December 2018, one identical *CaM* sequence (strain IHEM 18080, accession number MH644969) was deposited in GenBank by D’hooge et al. [42]. According to these authors, the position of this strain relative to *A. welwitschiae* and *A. niger* is not resolved, and they decided to provisionally name them *A. aff. welwitschiae*. Based on a concatenated tree (ITS, *BenA* and *CaM*), this strain was positioned next to *A. welwitschiae* with low support.

The concept of genealogical concordance has been widely used to recognise species boundaries, and has supported the description of new species [43,44,45,46,47,48,49,50]. As an example, recently, Frisvad et al. [51] revised the taxonomy of the *Aspergillus* section *Flavi* and with the help of GCPSR described eight new species. The GCPSR allows us to determine the limit of a species through a protocol based on the concordance/conflict of the different genealogies, thus reducing the subjectivity in recognising these limits. However, as pointed in Rintoul et al. [52], the limits of the species must be interpreted as explicit taxonomic hypotheses and must be tested with additional data, and/or based on other concepts.

### 3.3. RAPD Analysis

**Figure 3:**
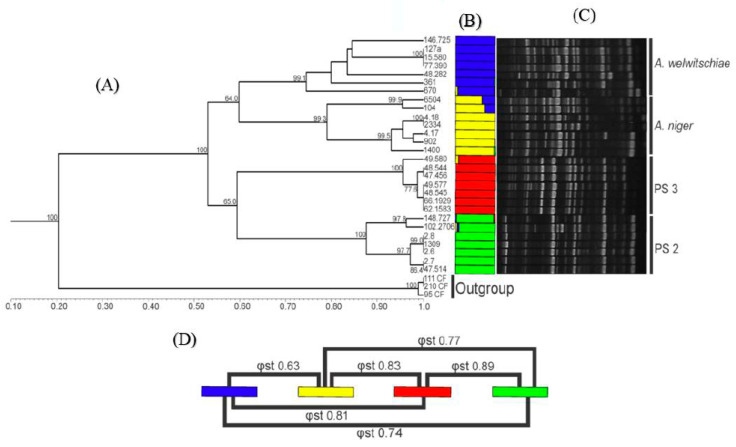
Cluster analysis based on Random Amplified Polymorphic DNA (RAPD) markers. PS2 = Phylogenetic species 2, PS3 = *Aspergillus vinaceus* sp. nov. Each colour corresponds to a genetic group (*K* = 4). (**A**) Unweighted Pair Group Method with Arithmetic Mean (UPGMA) dendrogram obtained using the Dice similarity coefficient using *A. carbonarius* strains as the outgroup. (**B**) Bayesian analysis (Structure bar plot). (**C**) RAPD profile representative, OPAM 07 primer. (**D**) Pairwise φst index.

A total of 92 reproducible RAPD bands were obtained from six random primers. The *dendrogram generated* by *UPGMA cluster analysis using the Dice similarity coefficient* was congruent to the results obtained using sequence data. Four well-defined groups were found, with one of them being composed of *A. niger* strains (GI), another being composed of *A. welwitschiae* strains (GII) and the two other groups formed by strains here recognised as belonging to PS2 (GIV) and PS3 (GIII) (Figure 3).

Several studies have used “*F*” statistic and its analogs as indicative of the degree of genetic structuring/differentiation between several groups of fungi [53,54,55,56,57,58,59,60]. In our study, the pairwise *φst* analysis corroborated the GCPSR findings. The values of *φst* for the comparisons among the four phylogenetic species ranged from 0.63 to 0.89 (Figure 3), values that can be interpreted as a very large differentiation [61]. Massi et al. [62] used the value of *Fst* as support to discriminate *Aspergillus nomius* from *Aspergillus pseudonomius*, two closely related species. According to the authors, values of *Fst* above 0.73 are uncommon for strains of the same species.

In the Bayesian clustering analysis, it was observed that the ideal number of clusters to allocate all strains was 4 (the best *K* value according to the model described by Evanno et al. [33]). As expected, the strains of the putative new species (PS2 and PS3) were designated as a group different from the groups of *A. niger* and *A. welwitschiae*, which are represented by a colour-coded bar graph shown in Figure 3. These findings corroborated with UPGMA analysis and with the GCPSR results.

The genotypic data (GCPSR and RAPD) obtained in the present study allows the validation of PS2 and PS3 as new phylogenetic species pertaining to the *A. niger* clade. Molecular approaches provide the largest number of variable characters for fungal taxonomy; currently, many phylogenetic species are being recognised. However, according to the International Code of Nomenclature for algae, fungi and plants, for the description of new species, differential phenotypic characters must be presented. Therefore, we decided to investigate the phenotypic traits of phylogenetic species 2 and 3.

### 3.4. Taxonomy

**Figure 4:**
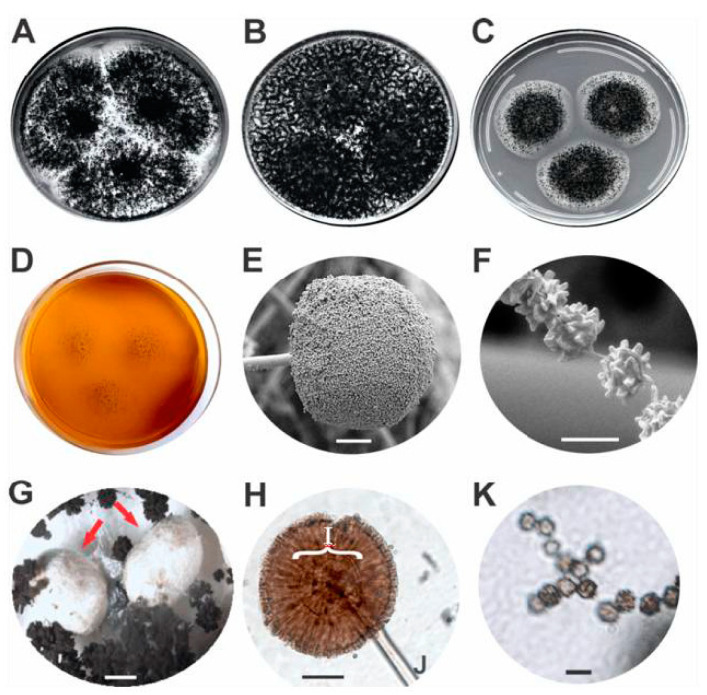
*Aspergillus vinaceus* sp. nov. MB 833399 (= ITAL 47.456, = IBT 35556): (**A**) Colonies on CYA (Czapek Yeast Autolysate agar), (**B**) colonies on YESA (Yeast Extract Sucrose Agar), (**C**) colonies on MEA (Malt Extract Agar), (**D**) colonies on CREA (Creatine Sucrose Agar). Scanning Electron Microscopy: (**E**) conidiophore (scale bar 50 µm), (**F**) conidia (scale bar 5 µm). Optical Microscopy: (**G**) sclerotia (scale bar 500 µm) were indicated using red arrows, (**H**) conidiophore, (**I**) vesicle, (**J**) stipe (scale bar 50 µm), (**K**) conidia (scale bar 5 µm).

A morphological analysis was carried out to establish a comparison between phylogenetic species found in GCPSR. No morphological differences were found among strains representing PS2, PS4 (*A. niger*) and PS1 (*A. welwitschiae*), however, the PS3 strains *differ* in *their morphology* *when compared to any of the other phylogenetic species analysed here.* The PS3 strains show reduced growth *on MEA medium* in relation to PS2, *A. niger* and *A. welwitschiae* strains (Table 2). The colonies of PS3 strains grown on CYA (25 °C) show white mycelial area staining with very sparse sporulation. PS3 strains have conidiophores (169 ± 23.1 μm), stipes width (18.3 ± 2.1 μm) and vesicles (68.2 ± 8.8 μm) of notably larger size in relation to the other species of the *A. niger* clade (Table 2). The conidia (4.6 ± 0.6 μm) varied from finely roughened to echinulate (Figure 4), while *A. niger* and *A. welwitschiae* conidia varied from smooth to rough. PS3 strains produce sclerotia, with ovoid shape, colour varying from white to cream, measuring on average 1200 ± 300 μm (Figure 4). Sclerotial development is an important prerequisite for sexual development in *Aspergillus* subgenus *Circumdati*, but only some species of *Aspergillus* section *Nigri* have been reported to produce sclerotia on well-known growth media. As shown by Frisvad et al. [63], no strains of *A. niger sensu* stricto produced sclerotia on conventional media. In certain strains of this species, sclerotium production may occur, but only when whole-raising or other plant parts are added to CYA medium (CYAR). *A. welwitschiae* did not produce sclerotia under any known conditions.

To conclude, the morphology data did not support the separation of PS2 from *A. niger* and *A. welwitschiae*; however, it is well-known that genetic changes can be observed before the fixation of the morphological and behavioural changes. In contrast, the PS3 can be diagnosed based on morphological characters and this phylogenetic species can also be framed in the morphological species concept.

In addition to morphological and DNA sequence features, it is strongly recommended to use secondary metabolites in species descriptions due to their high species specificity [64]. Secondary metabolite profiling has been used quite extensively for taxonomic purposes in *Aspergillus* section *Nigri*. Following the taxonomic schemes proposed by Samson et al. [5], we examined some strains (*n* = 10) of PS3 for secondary metabolite production on CYA and YESA by ultra-high-performance liquid chromatography using diode array detection (UHPLC-DAD). The strains produced acetyl-leucomelone, asperazine, funalenone, nigragillin, pyranonigrin A, tensidol B, malformins and very large amounts of a polar naphtho-gamma-pyrones (Fonsecin B, Aurasperones B, C and F) and the uncharacterised metabolites “HUTI” (Supplementary Figure S4) and “SURI” (Supplementary Figure S5).

As already presented, PS3 strains are molecularly most closely related to *A.* *niger* sensu stricto and *A. welwitschiae*. Because they may produce sclerotia on MEA, CYA and YESA media, which do not occur in strains of *A. niger* sensu stricto and *A. welwitschiae,* it was decided to perform the secondary metabolic characterisation in the sclerotia produced by the type strain ITAL 47.456 using HPLC-HRMS. The following strictly sclerotium-associated secondary metabolites were found in the strain ITAL 47.456: 14-Epi-hydroxy-10,23-dihydro-24,25-dehydroaflavinine; 10,23-Dihydro-24,25-dehydroaflavinine and 10,23-Dihydro-24,25-dehydro-21-oxo-aflavinine (Supplementary Table S2). Based on secondary metabolites profile, the strains of PS3 differ chemically from the other species of the *A. niger* clade (Table 3). It is important to note that in contrast to *A. niger* and *A. welwitschiae,* which are recognised as potentially producing species of ochratoxin A and/or fumonisins [65,66], none of the PS3 strains (*A. vinaceus* sp. nov.) were able to produce fumonisins and/or ochratoxins.

Based on the agreement between multi-locus species delimitation results, macro- and micro-morphological characters (Figure 4), genetic diversity measured by RAPD and secondary metabolites profile, the phylogenetic species 3 (PS3) is proposed in this report to be a new species in the *Aspergillus* subgenus *Circumdati* section *Nigri*, named *Aspergillus vinaceus.*

Since *A. vinaceus* sp. nov. is closely related to *A. welwitschiae* and *A. niger*, which have huge industrial potential and biotechnological relevance, but, in contrast is not able to produce fumonisins and/or ochratoxins, their description opens new avenues for biotechnological potential research.

Taxonomy: Aspergillus vinaceus Ferranti, Iamanaka, Frisvad, Puel & Silva, sp. nov. (Figure 4)

MycoBank number: MB 833399.

Etymology: “*vinaceus*” is referred to wine-grapes on which this fungus was collected.

Holotype: ITAL 47.456 (T) (= IBT 35556), a freeze-dried culture in Coleção de Cultura Tropical (Campinas, Brazil) is designated as the holotype of *A. vinaceus*. It was isolated from the surface of grape berries (*Vitis labrusca*), Petrolina, Brazil, 2015, by Ferranti L.S. and Iamanaka, B.T.

Other strains examined and deposited strains in the IBT collection: ITAL 48.544 (= IBT 35557), ITAL 66.1929 (= IBT 35561), ITAL 48.545 (= IBT 35558).

Colony diameters: 7 days (in mm): CYA 25 °C 64–66, CYA 37 °C 60–63.5, CYA 42 °C 23–30, MEA 25 °C 35–39, YESA 25 °C 65–67.

Description: Colony attaining a diameter of 64–66 mm on CYA after 7 days at 25 °C, 60–63.5 mm at 37 °C and 23–30 mm at 42 °C, white mycelium, abundant sporulation, clear and abundant exudate. On YESA, colony of 65–67 mm diameter after 7 days at 25 °C, growth pattern similar to that on CYA at 25 °C except more abundant sporulation. On MEA, colony of 35–39 mm diameter after 7 days at 25 °C, moderate sporulation, white mycelia surrounding a central sporulation area. On CREA, colony small, poor sporulation but good acid production. Conidial heads biseriate, radiate, commonly splitting into columns, up to 200 μm in diameter on MEA. Stipes 1300–1800 μm long, 16–21 μm wide, hyaline, smooth. Vesicles 63–75 µm in diameter, globose to sub-globose. Metulae 20–40 µm. Phialides 15–27 µm, covering entire vesicle. Conidia 3–5.5 μm, globose to sub-globose, brown to black, finely roughened to echinulate. Sclerotia 900–1500 μm, white to cream, produced on CYA, YESA and MEA at 25 °C after 7 days but without sclerotial production at 37 and 42 °C on CYA.

Secondary Metabolites:

From whole fungal culture (UHPLC-DAD): Acetyl-leucomelone, Asperazine, Aurasperone B, Aurasperone C, Aurasperone F, Fonsecin B, Funalenones, HUTI, Malformin A1, Malformin C, Nigragillin, Pyranonigrin A, SURI, Tensidol B.

From sclerotium extracts (HPLC-HRMS):14-Epi-hydroxy-10,23-dihydro-24,25-dehydroaflavinine; 10,23-Dihydro-24,25-dehydroaflavinine; 10,23-Dihydro-24,25-dehydro-21-oxo-aflavinine, Aurasperone B, Aurasperone C, Aurasperone F, Fonsecin, Fonsecin B, Funalenone, Malformins A1, A2 e A4, Malformin C, Nigragillin, Pyrophen, Tensidol A, Tensidol B (Supplementary Table S2).

Barcode: ITS = MN575692, *BenA*  =  MN583579, *CaM*  =  MN583580, *RPB2* = MN583581.

Notes: *Aspergillus vinaceus* is molecularly related to *A. welwitschiae* and *A. niger,* but clearly morphologically distinguishable. In CYA, colonies of *A. vinaceus* have a white prominent mycelial area. In MEA, the growth of *A. vinaceus* is significantly lower than the other species of the clade *A. niger.* In addition, it produces very large (900–1500 μm) white to cream colour sclerotia on MEA, CYA and YESA media, which do not occur in strains of *A. niger* and *A. welwitschiae*. Microscopically, the conidiophore, stipe width and vesicle are substantially larger than in the other closely related species. The conidia are echinulate rather than with black bars, as in *A. niger* and *A. welwitschiae*. *A. vinaceus* produces a spectrum of secondary metabolites which is different from *A. niger* and *A. welwitschiae* (Table 3).

## Figures and Tables

**Table 1:** Polymerase Chain Reaction (PCR) conditions for *CaM, BenA* and *RPB2 loci.*

			35 Cycles			
		1 min	45 s	45 s	1 min *	5 min
	Primers	Initial Denaturation	Denaturation	Annealing	Extension	Final Extension
Thermal program	*CaM* (Hong et al. [15])	95 °C	94 °C	55 °C	72 °C	72 °C
	*BenA* (Glass and Donaldson [17])	95 °C	94 °C	60 °C	72 °C	72 °C
	*RPB2* (this study)	95 °C	94 °C	58 °C	72 °C	72 °C
PCR conditions	*Taq* DNA polymerase	1 U				
	PCR buffer	1×				
	Primer	0.4 µM—each primer				
	dNTP	0.2 Mm				
	MgCl_2_	1.5 Mm				
	DNA template	10 ng				

* 1 min and 30 s for *RPB2 locus.*

**Table 2:** Morphological characteristics of the phylogenetic species (PS) found in the *A. niger* clade.

			Growth Rate (mm/7 days)			
		Temperature	PS1 (*A. welwitschiae*)	PS2	PS3 (*A. vinaceus* sp. nov.)	PS4 (*A. niger*)
Macromorphology		25 °C	63.3 ± 0.9	63 ± 0.8	65.2 ± 1.2	64.7 ± 0.5
	CYA	37 °C	62.3 ± 0.8	61 ± 0.3	63.2 ± 0.4	62.3 ± 1.2
		42 °C	34 ± 4.3	39 ± 0.2	23.6 ± 1.2	28 ± 0.8
	MEA	25 °C	54.2 ± 5.3	50 ± 0.4	38.2 ± 1.2	58.7 ± 4.7
	YESA	25 °C	65.7 ± 2.3	68 ± 0.7	67.2 ± 0.8	67 ± 1.4
	Sclerotia		Absent	Absent	White to cream	Absent
	Conidia colour		Black	Black	Black	Black
	Colour in mycelial area		Black	Black	White	Black
Micromorphology *	Vesicle shape		Globose to sub-globose	Globose to sub-globose	Globose to sub-globose	Globose to sub-globose
	Conidial ornamentation		Smooth to roughened	Smooth to finely roughened	Finely roughened to echinulate	Smooth to finely roughened
	Vesicle size		40.5 ± 5.2	45.8 ± 4.1	68.2 ± 8.8	41.7 ± 3.2
	Conidiophore size		76.5 ± 12.7	86.6 ± 11.9	169 ± 23.1	82.2 ± 12.8
	Conidial size		4.1 ± 0.6	4 ± 0.6	4.6 ± 0.6	3.8 ± 0.5
	Stipe width		12.8 ± 1.6	14.6 ± 1.1	18.3 ± 2.1	12.4 ± 0.8

* Micromorphological analysis was performed in MEA medium, and the unit of measurement is micrometres (μm). Czapek Yeast Autolysate agar (CYA); Malt Extract Agar (MEA); Yeast Extract Sucrose Agar (YESA); degree Celsius (°C).

**Table 3:** Principal secondary metabolites produced by *Aspergillus niger* clade species.

Fungal Sample	Metabolites	*Aspergillus niger* Clade Species		
		*A. vinaceus* sp. nov. (PS3)	*A. niger*	*A. welwitschiae*
Whole fungal culture *	Acetyl-leucomelone	+	+	+
	Asperazine	+	-	-
	Aurasperone B	+	+	+
	Aurasperone C	+	+	+
	Aurasperone F	+	+	+
	Fonsecin B	+	+	+
	Fumonisin B2	-	+	+
	Fumonisin B4	-	+	+
	Fumonisin B6	-	+	+
	Funalenones	+	+	+
	HUTI	+	+	+
	Malformin A1	+	+	+
	Malformin C	+	+	+
	Nigragillin	+	+	+
	Ochratoxin A	-	+	+
	Pyranonigrin A	+	+	+
	SURI	+	+	+
	Tensidol B	+	+	+
Sclerotia extracts **	14-Epi-hydroxy-10,23-dihydro-24,25-dehydroaflavinine	+	-	-
	10,23-Dihydro-24,25-dehydroaflavinine	+	-	-
	10,23-Dihydro-24,25-dehydro-21-oxo-aflavinine	+	-	-
	Aurasperone B	+	-	-
	Aurasperone C	+	-	-
	Aurasperone F	+	-	-
	Fonsecin	+	-	-
	Fonsecin B	+	-	-
	Funalenone	+	-	-
	Malformins A1, A2 and A4	+	-	-
	Malformin C	+	-	-
	Nigragillin	+	-	-
	Pyrophen	+	-	-
	Tensidol A	+	-	-
	Tensidol B	+	-	-

* Grown on CYA and YESA media. ** Sclerotia produced on MEA medium.

## Supplementary Materials

The following are available online at https://www.mdpi.com/2309-608X/6/4/371/s1: Table S1: Listing of the strains and distribution of haplotypes found in this study. Table S2: Secondary metabolites data of *Aspergillus vinaceus* sp. nov. sclerotia, produced in MEA medium, detected by liquid chromatography coupled to high-resolution mass spectrometry (HPLC-HRMS). Figure S1: Alignment of partial *RPB2* gene sequences of *A. niger* aggregate. The boxes marked by arrows indicate the annealing site of the ANRP primer pair. * ANRP1-F or ANRP2-R designed in this study. Figure S2: Maximum parsimony tree (MR 50%) of *A. niger* clade species using the *loci CaM, BenA* and *RPB2*. Genealogical Concordance criterion: The lineages that occur in most of the single *loci* are highlighted in coloured boxes, and these lineages were identified as independent evolutionary lineages. Bootstrap values (BP) and/or posterior probabilities values (PP) higher than 70% and 0.70 respectively, are shown. *A. carbonarius* is the outgroup. Figure S3: Maximum parsimony tree (semi-strict consensus) of *A. niger* clade species using three-locus (*CaM, BenA* and *RPB2*) phylogenies. Genealogical non-discordance criterion: The lineages that were well-supported by at least one *locus* but not contradicted by any other locus are identified as independent evolutionary lineages (coloured boxes). Bootstrap values (BP and/or posterior probabilities values (pp) higher than 70% and 0.70 respectively, are shown. *A. carbonarius* is the outgroup. Figure S4: UV spectrum of metabolite “HUTI” (RT: retention time, RI: retention index, log A: logarithm of the area of the peak in the chromatogram). Figure S5: UV spectrum of metabolite “SURI” (RT: retention time, RI: retention index, log A: logarithm of the area of the peak in the chromatogram).
