# Supplementary material for: Diversity within Aspergillus niger Clade and Description of a New Species: Aspergillus vinaceus sp. nov
Source: J Fungi (Basel). 2020 Dec 17;6(4):371. doi: 10.3390/jof6040371 (PMC7767288; doi:10.3390/jof6040371)
Supplement: Supplementary file 1 [file jof-06-00371-s001.zip › Supplementary materials/Supplementary Fig. S2.docx]

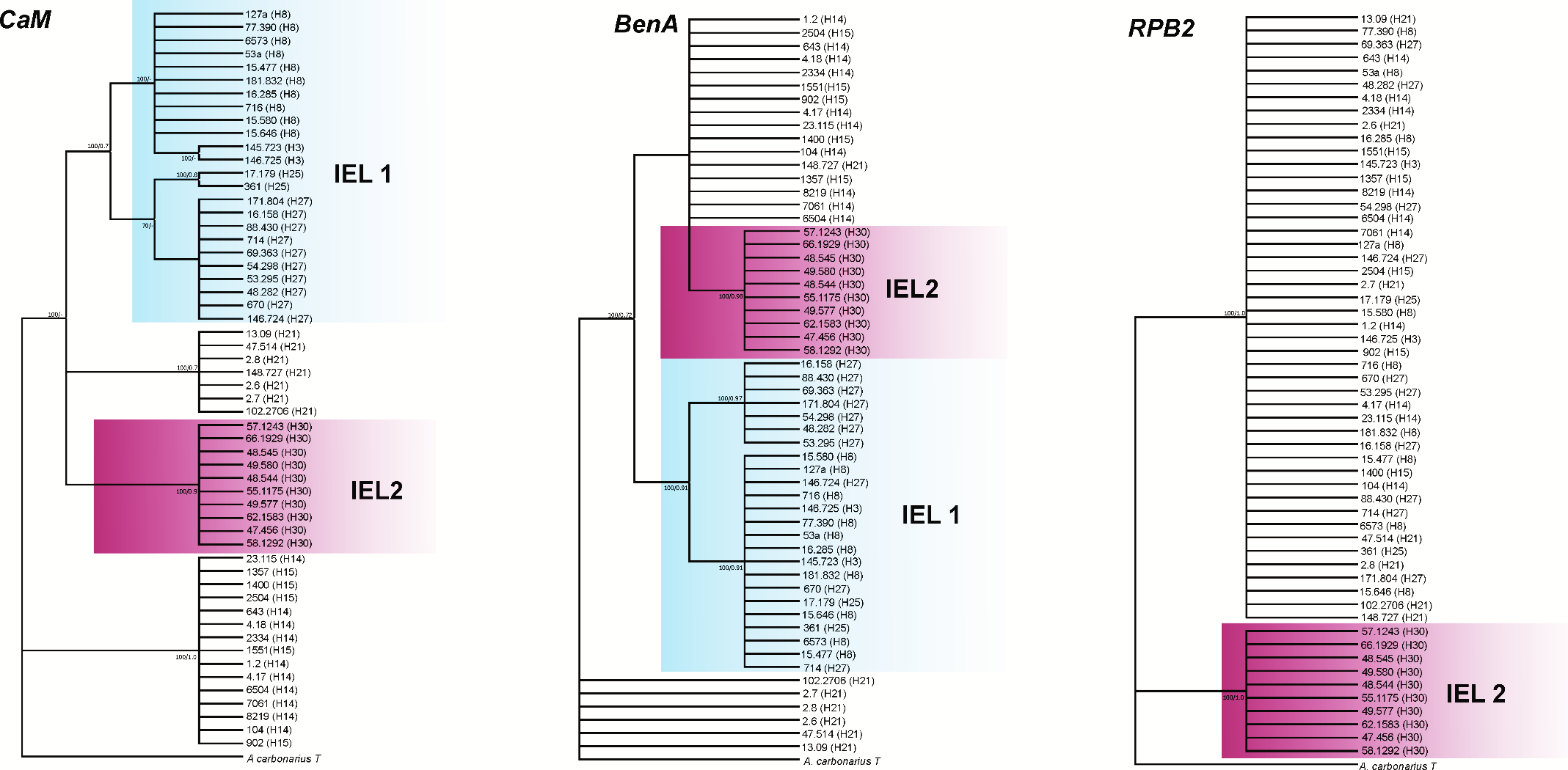
 **Supplementary Figure S2.** Maximum parsimony tree (MR 50%) of *A. niger* clade species using the *loci* *CaM, BenA*, and *RPB2*. Genealogical Concordance criterion: The lineages that occur in most of the single *loci* are highlighted in coloured boxes, and these lineages were identified as independent evolutionary lineages. Bootstrap values (BS) and/or posterior probabilities values (pp) higher than 70% and 0.70, respectively, are shown. *A. carbonarius* is the outgroup.
