# Supplementary material for: Diversity within Aspergillus niger Clade and Description of a New Species: Aspergillus vinaceus sp. nov
Source: J Fungi (Basel). 2020 Dec 17;6(4):371. doi: 10.3390/jof6040371 (PMC7767288; doi:10.3390/jof6040371)
Supplement: Supplementary file 1 [file jof-06-00371-s001.zip › Supplementary materials/Supplementary Fig. S3.docx]

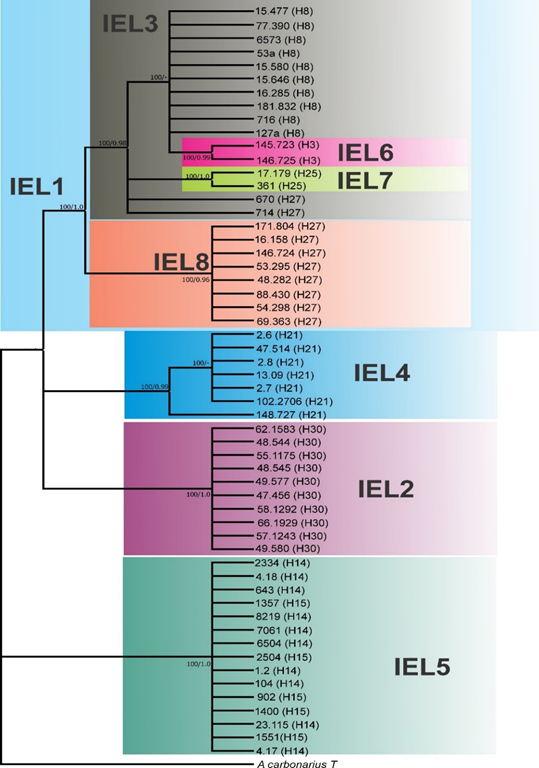


**Supplementary Figure S3**. Maximum parsimony tree (semi-strict consensus) of *A. niger* clade species using three-locus (*CaM, BenA*, and *RPB2*) phylogenies. Genealogical non-discordance criterion: The lineages that were well supported by at least one *locus* but not contradicted by any other locus are identified as independent evolutionary lineages (coloured boxes). Bootstrap values (BS) and/or posterior probabilities values (pp) higher than 70% and 0.70, respectively, are shown. *A. carbonarius* is the outgroup.
