# Supplementary material for: Diversity within Aspergillus niger Clade and Description of a New Species: Aspergillus vinaceus sp. nov
Source: J Fungi (Basel). 2020 Dec 17;6(4):371. doi: 10.3390/jof6040371 (PMC7767288; doi:10.3390/jof6040371)
Supplement: Supplementary file 1 [file jof-06-00371-s001.zip › Supplementary materials/Supplementary Fig. S5.docx]

**Supplementary Figure S5**: UV spectrum of metabolite “SURI” (RT: retention time, RI: retention index, log A: logarithm of the area of the peak in the chromatogram).
