# Supplementary material for: Diversity within Aspergillus niger Clade and Description of a New Species: Aspergillus vinaceus sp. nov
Source: J Fungi (Basel). 2020 Dec 17;6(4):371. doi: 10.3390/jof6040371 (PMC7767288; doi:10.3390/jof6040371)
Supplement: Supplementary file 1 [file jof-06-00371-s001.zip › Supplementary materials/Supplementary Fig. S1.docx]

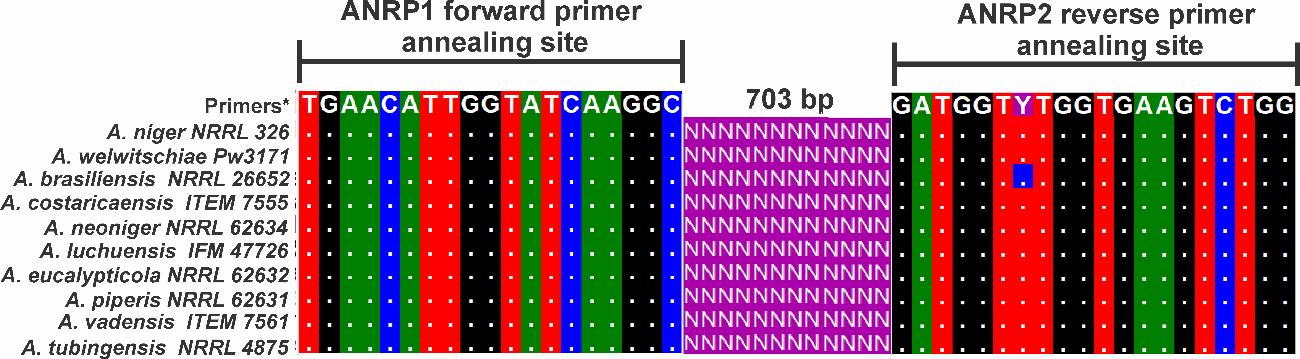


**Supplementary Figure S1**. Alignment of partial *RPB2* gene sequences of *A. niger* aggregate. The boxes marked by arrows indicate the annealing site of the ANRP primer pair. * ANRP1-F or ANRP2-R designed in this study.
