# Supplementary material for: Diversity within Aspergillus niger Clade and Description of a New Species: Aspergillus vinaceus sp. nov
Source: J Fungi (Basel). 2020 Dec 17;6(4):371. doi: 10.3390/jof6040371 (PMC7767288; doi:10.3390/jof6040371)
Supplement: Supplementary file 1 [file jof-06-00371-s001.zip › Supplementary materials/Supplementary Table S2.docx]

| **Metabolite** | **Elemental Composition** | **Ion** | **Retention Time (min)** | **Measured mass** | **Error (ppm)** | **MS/MS spectra** | **Normalized level** |
| --- | --- | --- | --- | --- | --- | --- | --- |
| 14-Epi-14-hydroxy-10,23-dihydro-24,25-dehydroaflavinine | C28H39NO2 | [M+H]+ | 40.18 | 422,30467 | (-1.601) | 422 ---> 404 (100), 291 (22), 386 (18), 231 (9), 130 (6) | 8,12E+07 |
| 10,23-Dihydro-24,25-dehydroaflavinine | C28H39NO | [M+H]+ | 42.65 | 406,3098 | (-1.579) | 406 ---> 388 (100), 160 (30), 158 (18), 350 (12), 229 (10), 130 (è), 306 (8), 271 (6) | 2,51E+07 |
| 10,23-Dihydro-24,25 dehydro-21-oxo-aflavinine | C28H38NO2 | [M+H]+ | 37.76 | 420,28902 | (-1.632) | 420 ---> 402 (100), 384 (9), 285 (7), 374 (4), 158 (4), 172 (3), 372 (3), 303 (3), 130 (3) | 2,41E+07 |
| Aurasperone B | C32H30O12 | [M+H]+ | 31.58 | 607,17987 | (-1.857) | 607 ---> 589 (100), 549 (42), 565 (31), 505 (28), 533 (12) | 1,19E+08 |
| Aurasperone C | C31H28O12 | [M+H]+ | 26.20 | 593,16343 | (-3.241) | 593 ---> 575 (100), 535 (50), 551 (50), 493 (8), 551 (7), 509 (7), 517 (6) | 1,90E+07 |
| Aurasperone F | C31H26O11 | [M+H]+ | 29.16 | 575,1528 | (-3.447) | 575 ---> 517 (100), 557 (42), 491 (18), 533 (14), 502 (6) | 3,79E+06 |
| Fonsecin | C15H14O6 | [M+H]+ | 12.53 | 291,08526 | (-3.623) | 291 ---> 249 (100), 233 (40), 273 (20), 207 (6), 231 (2) | 5,76E+05 |
| Foncesin B | C16H1606 | [M+H]+ | 22.41 | 305,10081 | (-3.752) | 305 ---> 263 (100), 247 (58), 287 (25), 221 (6) | 1,34E+06 |
| Funalenone | C15H12O6 | [M+H]+ | 16.47 | 289,06961 | (-3.648) | 289---> 274 (100), 243 (2), 271 (1) | 2,28E+05 |
| Malformin A1 | C23H39O5N5S2 | [M+H]+ | 26.74 | 530,24546 | (-2.032) | 530 ---> 417 (100), 485 (42), 502 (22), 372 (9), 304 (6), 512 (6), 487 (4), 389 (3) | 7,70E+07 |
| Malformin A2 | C22H37N5O5S2 | [M+H]+ | 22.99 | 516,22912 | (-3.423) | 516 ---> 417 (100), 471 (76), 488 (38), 403 (26), 496 (11), 372 (8) 304 (7), 443 (6), 358 (5) | 1,07E+07 |
| Malformin A4 | C22H37N5O5S2 | [M+H]+ | 21.65 | 516,22903 | (-3.598) | 516 ---> 403 (100), 471 (50), 488 (26), 498 (10), 358 (8), 417 (6), 304 (5),185 (1), 199, 171 | 3,89E+06 |
| Malformin C | C23H39O5N5S2 | [M+H]+ | 28.08 | 530,24553 | (-1.899) | 530 ---> 417 (100), 485 (78), 502 (46), 372 (18), 304 (7), 512 (8), 457 (5) | 8,56E+07 |
| Nigragillin | C13H22N2O | [M+H]+ | 1.91 | 223,17985 | (-2.867) | 223 --->129 (100), 166 (7), 995 (7) | 4,44E+06 |
| Pyrophen | C16H17NO4 | [M+H]+ | 13.24 | 288,12205 | (-3.417) | 288 --->246 (100), 228 (7), 210 (5), 229 (3), 270 (2) | 5,29E+06 |
| Tensidol A | C13H11NO3 | [M+H]+ | 13.56 | 230,0803 | (-3.780) | 230 ---> 213 (100) | 1,12E+05 |
| Tensidol B | C18H17NO6 | [M+H]+ | 21.79 | 344,11232 | (-1.565) | 344 ---> 230 (100), 326 (6), 213 (1) | 4,06E+06 |

**Supplementary Table S2.** Secondary metabolites data of *Aspergillus vinaceus* sp. nov. sclerotia, produced in MEA medium, detected by liquid chromatography coupled to

high-resolution mass spectrometry (HPLC-HRMS).
